# Ethical deliberations about involuntary treatment: interviews with Swedish psychiatrists

**DOI:** 10.1186/s12910-015-0029-5

**Published:** 2015-05-28

**Authors:** Manne Sjöstrand, Lars Sandman, Petter Karlsson, Gert Helgesson, Stefan Eriksson, Niklas Juth

**Affiliations:** 1 Stockholm Centre for Healthcare Ethics, Department of Learning, Informatics, Management and Ethics, Karolinska Institutet, Stockholm, Sweden and Center for Bioethics, Harvard Medical School, Boston, MA USA; 2 Academy for care, work-life and welfare, University College of Borås, Borås, Sweden; 3 National Centre for Priority Setting in Health-care, Linköping University, Linköping, Sweden; 4 Centre for Research Ethics and Bioethics, Department of Public Health and Caring Sciences, Uppsala University, Uppsala, Sweden; 5 Stockholm Centre for Healthcare Ethics, Department of Learning, Informatics, Management and Ethics, Karolinska Institutet, Stockholm, Sweden

**Keywords:** Psychiatry, Bioethics, Personal autonomy, Paternalism, Coercion, Involuntary commitment

## Abstract

**Background:**

Involuntary treatment is a key issue in healthcare ethics. In this study, ethical issues relating to involuntary psychiatric treatment are investigated through interviews with Swedish psychiatrists.

**Methods:**

In-depth interviews were conducted with eight Swedish psychiatrists, focusing on their experiences of and views on compulsory treatment. In relation to this, issues about patient autonomy were also discussed. The interviews were analysed using a descriptive qualitative approach.

**Results:**

The answers focus on two main aspects of compulsory treatment. Firstly, deliberations about when and why it was justifiable to make a decision on involuntary treatment in a specific case. Here the cons and pros of ordering compulsory treatment were discussed, with particular emphasis on the consequences of providing treatment *vs.* refraining from ordering treatment. Secondly, a number of issues relating to background factors affecting decisions for or against involuntary treatment were also discussed. These included issues about the Swedish Mental Care Act, healthcare organisation and the care environment.

**Conclusions:**

Involuntary treatment was generally seen as an unwanted exception to standard care. The respondents’ judgments about involuntary treatment were typically in line with Swedish law on the subject. However, it was also argued that the law leaves room for individual judgments when making decisions about involuntary treatment. Much of the reasoning focused on the consequences of ordering involuntary treatment, where risk of harm to the therapeutic alliance was weighed against the assumed good consequences of ensuring that patients received needed treatment. Cases concerning suicidal patients and psychotic patients who did not realise their need for care were typically held as paradigmatic examples of justified involuntary care. However, there was an ambivalence regarding the issue of suicide as it was also argued that risk of suicide in itself might not be sufficient for justified involuntary care. It was moreover argued that organisational factors sometimes led to decisions about compulsory treatment that could have been avoided, given a more patient-oriented healthcare organisation.

**Electronic supplementary material:**

The online version of this article (doi:10.1186/s12910-015-0029-5) contains supplementary material, which is available to authorized users.

## Background

Whether it is justifiable to treat patients against their will and, if so, when, is a central question in medical ethics and law. Respect for autonomy is a central principle in contemporary healthcare ethics [1]. A common assertion is that respect for autonomy in healthcare implies, at a minimum, that patients should not be coerced or manipulated into treatment if they are capable of making autonomous decisions about their care and treatment [2]. In recent bioethical debate, the idea that autonomy is something valuable that should be protected and promoted has become influential [3]. The idea is of particular interest in the context of involuntary psychiatric treatment, where it can be argued that involuntary treatment could be justified in order to restore autonomy [4].

**Table 1:** Themes, categories, and subcategories extracted from the interviews

Theme	Category	Subcategory
The pros and cons of ordering involuntary treatment	Fulfilling the patients’ need of care	Preventing suicide
		Providing necessary psychiatric treatment
		Ensuring treatment of somatic disorders
		Protecting the patient from social harms
	Promoting autonomy	Restoring autonomous ability
		Promoting well-reasoned decisions
		Respecting the patient’s presumed will
	Safeguarding third party interests	Preventing harm to others
		Relieving relatives of responsibility
	Reasons against involuntary treatment	Involuntary treatment as an unwanted exception
		Avoiding disruption of trust
		Avoiding direct harms of coercion
		Respecting self-determination within limits
Circumstances affecting decisions about involuntary treatment	The patient’s social circumstances	Accepting the possibility of rational suicides
	Legal influence	Legal demands
		Interpreting the law
	The possibility of informal coercion	Restricting options
		Using the law to make the patient accept "voluntary" treatment
	Healthcare deficiencies	Inadequate care environment
		Inadequate resources

Laws on involuntary treatment in psychiatry typically call for treatment of psychiatric disorders based on concerns relating to the severity of the patient’s condition, need for treatment, and danger to self or others [5–7]. Lack of decision-making capacity, which typically is defined in terms of abilities for understanding, appreciation, reasoning and communication [8], is generally not held as a necessary criterion for involuntary psychiatric treatment [5–7]. However, studies have shown that also patients with serious psychiatric disorders may have decision-making capacity according to standard criteria [9–11].

In recent bioethical debate, current legal standards have been criticised, and it has been argued that involuntary treatment is only justified in cases where patients are incapable of autonomous decision-making [4, 12–14], and primarily for the sake of their own needs, not for the sake of protecting others from harm [2, 12–15]. There is also a discussion on the use of advance directives in psychiatry [16, 17]. However, there are controversial issues relating to the extension of such directives, for instance whether patients should have a right to reject future care [18, 19].

### Empirical background

There is no conclusive data on the effectiveness of involuntary treatment with regard to treatment outcomes [20–23]. Quantitative as well as qualitative studies on patients’ attitudes to involuntary treatment report mixed findings. Although many patients believe involuntary treatment to be justified and necessary, follow-up studies suggest that a substantial number of patients disapprove of having been subjected to involuntary treatment, and negative experiences relating to restriction of freedom as well as violations of personal integrity are reported [24–30]. It is also important to note that formal legal status does not always correspond to what patients experience as coercion; hence patients may report being subjected to coercive measures despite formally being under voluntary treatment [24, 31, 32].

In a survey of American psychiatrists’ views about involuntary treatment, respondents held danger to self or others and grave disability as the primary reasons for involuntary treatment [33]. Surveys of psychiatrists’ views in Norway and Sweden have shown similar results [34, 35]. A recent qualitative study in a Norwegian setting shows that considerations relating to beneficence and protecting patients from harm were more important to clinicians than considerations about patients’ ability to make autonomous treatment decisions. The study also argues that the clinicians’ decisions about involuntary admission were influenced by extra-legal factors, such as patients’ needs and attitudes toward treatment, expected consequences of involuntary admission, the psychiatric services’ follow-up routines, and the patients’ social circumstances [36].

The present study is a contribution to the discussion about the ethics of involuntary treatment in psychiatry. The article presents results from in-depth interviews with eight Swedish psychiatrists, aiming to explore the psychiatrists’ ethical reasoning regarding involuntary psychiatric treatment. Additionally, the article discusses the psychiatrists’ experiences and reasoning in relation to contemporary bioethical discussion about involuntary treatment in psychiatry.

### The Swedish Mental Health Act

In Sweden, patients have a basic right to reject proposed medical treatment and procedures. However, the right to reject care can under certain conditions be restricted, most importantly in cases of serious psychiatric disorder and in cases of certain infectious diseases [37, 38].

The Swedish law on involuntary psychiatric treatment specifies three necessary conditions for involuntary admission and treatment: (1) the patient has a serious psychiatric disorder, (2) the patient has an imperative need of psychiatric care, and (3) the patient refuses such care or is deemed incapable of making a decision on the subject [38]. The preliminary decision about involuntary admission can be made by any licensed physician. The decision, however, must be confirmed by a specialist in psychiatry within 24 h of the patient’s admittance to a psychiatric hospital. The decision is to be tried by a court of law if the patient demands it, or if the involuntary treatment period lasts for over four weeks [38, 39]. When a patient is admitted involuntarily, he or she is not allowed to leave the ward without permission from the attending psychiatrist, and forced treatment (injections), seclusion from other patients, and physical restraints (bed straps) can be ordered. In acute circumstances, decisions about forced treatment and restraints (typically in cases of severe agitation) can be made directly after the initial decision about involuntary admission. After a separate court decision, involuntary treatment may be continued at an outpatient facility after discharge from the hospital.

The notion of ‘serious psychiatric disorder’ typically concerns psychotic disorders, severe depressions, or manic states with psychotic symptoms. However, the law does not specify which disorders could be included. Hence, other psychiatric disorders, for instance severe depressions or personality disorders could be labelled as a serious psychiatric disorder if the symptoms are grave enough. Moreover, the law does not predicate any special kind of genesis for a disorder to be labelled as psychiatric – it is the manifest symptoms that count. The second criterion, ‘imperative need of psychiatric care’, applies to situations where grave consequences would follow for the patient (or in certain cases third parties) if treatment were not given. This refers not only to life-threatening situations or situations where there is risk of bodily harm to the patient or to others but may also include other kinds of harms, most importantly situations where the patient’s psychiatric condition will deteriorate without proper treatment [39]. However, the first criterion may be interpreted in the light of the second criterion. Hence, if a patient has a psychiatric disorder and is assessed to have an imperative need of inpatient psychiatric treatment, the patient’s disorder would typically qualify as serious.

The third requirement says that in cases where a patient fulfils the two first above-mentioned criteria but accepts treatment, involuntary treatment can still be ordered if the patient is deemed incapable of making a decision about his or her care. However, lack of capacity is not a necessary requirement for involuntary treatment and the notion of decision-making capacity is not defined in Swedish healthcare legislation.

With regard to somatic healthcare, there is no law permitting involuntary admission or treatment except in certain cases of infectious diseases [37]. Regulations from the National Board of Health and Welfare require assessment of decision-making capacity of patients who wish to reject life-supporting treatments, but the extent to which treatment can be given against the will of patients who lack adequate capacity is unclear [40]. Psychiatric patients with life-threatening somatic conditions may, however, be treated involuntary for their medical condition if the requirements regarding psychiatric involuntary treatment are satisfied [39].

## Methods

### Respondents

The respondents were recruited continuously during the project, with the aim of covering views and experiences from psychiatrists with different backgrounds and experiences (so-called purposive sampling) [41]. Participants were identified because of their experience and expertise, partly through previously established connections with members of the research team and partly through chain referral. All respondents were working in the Greater Stockholm area at the time of the interview, but several of them had previously worked in other parts of Sweden. The respondents were 30–68 years of age. Five respondents were specialists in general psychiatry, two were also specialists in forensic psychiatry, and two were working in addiction psychiatry. Three of the respondents were residents in general psychiatry, in the middle or at the end of their residencies. Four respondents were female and four were male.

The initial request for participation was sent by e-mail with information about the study, its purpose and methods. Information was then repeated in written and verbal form at the beginning of the interview. All respondents were informed that participation was voluntary and could be withdrawn at any time without further explanation. All respondents whom we established contact with decided to take part and no participants withdrew consent. One interview had to be cancelled due to scheduling difficulties. The study was approved by the Ethical Review Board in Stockholm (Dnr 2011/114-31).

### Interviews

The interviews were conducted in Swedish and were based on nine main open-ended questions (see Additional file 1), with the possibility of qualifying follow-up questions depending on the course of the interview. The focus of the interviews was conceptions of patient decision-making capacity, its relationship to psychiatric disorders, and reasoning and experiences concerning decisions about involuntary treatment. The participants were encouraged to use anonymised cases from their own clinical experience to elaborate on the questions. The interviews lasted about 1–1.5 h and were recorded with a digital voice recorder and transcribed verbatim. The number of interviews was not predetermined. Since the interviews were rich in data, we decided that our exploratory aim was achieved after eight interviews. The results concerning decision-making capacity are presented in a separate paper [42].

### Data analysis

The eight interviews were analysed using descriptive qualitative content analysis following Sandelowski [43]. The analysis was done inductively, without pre-set categories. Initially the text was read repeatedly to get an overall impression of the content. Next, meaning units, phrases expressing thoughts relating to the overall research questions, were identified in the text. Meaning units expressing the same idea were then sorted together in sub-categories that were organised into categories [43–45]. The categories about involuntary treatment were organised into two sections. The first section, covering reasoning regarding compulsory treatment, describes arguments for and against involuntary psychiatric treatment presented by the respondents in relation to cases. The second section of categories concerns the circumstances in which decisions about involuntary psychiatric treatment are made.

## Results

### The pros and cons of ordering involuntary treatment

In the first section, the respondents' reasoning regarding the pros and cons of ordering involuntary treatment are presented (Table 1).

#### Fulfilling the patients’ need of care

The most important reason stated for ordering involuntary treatment was the patients’ perceived need of care and treatment. Firstly, this was seen as necessary in cases where patients refused care and there was a risk of suicide. Secondly, involuntary treatment was seen as justified in cases where seriously ill patients refused treatment that was seen as essential for improving their condition, both psychiatric and somatic. Thirdly, also protection from social harms was seen as a viable reason for involuntary treatment.

##### Preventing suicide

Risk of suicide was generally raised as a major factor when deciding about compulsory treatment. One of the respondents recalled a case where a patient with a complicated background, but no established psychiatric diagnosis, had issued a suicide threat in a letter to his counsellor. When they met, the patient was calm and did not display any severe psychiatric symptoms.

*And this was one of the hardest cases. Because here is a person who at first sight seems just like anyone else. But who I know has this background and had expressed suicidal thoughts in this letter.* […] *After much hesitation I made the decision about involuntary admission after all. It was hard, but with this suicide business*…

-Specialist in psychiatry, female.

Another respondent told of a case in which he had respected a patient’s rejection of medical treatment for a depression. When asked what could have changed his mind so that he instead would have decided to admit the patient involuntarily, he replied:

*If she had planned a suicide attempt later the same week, then I couldn’t have waited.*

-Resident in psychiatry, male.

##### Providing necessary psychiatric treatment

The other typical example of involuntary treatment being considered justified was when patients rejected a treatment deemed necessary. The typical example concerned patients with psychotic disorders.

*There are many people whose apartments may be in total disarray and everything is messed up, but whom you do not see as mentally disordered. But if you have a chronically psychotic patient in that situation, then it is a sign that things are pretty serious. He cannot manage his everyday life anymore, and that is a sign that he needs care.*

-Specialist in psychiatry, male.

One respondent argued that it is sometimes necessary to give treatment involuntarily in order to show the patients that their condition can improve through medication.

*And you see that you have to medicate, at least in order to show that you can stop this. That, at least, you make some effort so that the person will not lose their entire life.*

-Specialist in psychiatry, female.

##### Ensuring treatment of somatic disorders

One more reason mentioned was need of care for somatic conditions. Here it was argued that patients with psychiatric disorders who mismanaged their somatic disorder could be involuntarily admitted to treat their somatic disorders.

*Then, if he [the patient] has a serious psychiatric disorder and doesn’t understand the seriousness of his somatic condition, I think that you have the right to breach his autonomy.*

-Specialist in psychiatry, male.

This, however, was seen as controversial and, in line with the law, typically only seen as justifiable in severe cases.

*But, I also realise that… well… that you are on thin ice, and well exactly… Where should you draw the line? Should you, like, be able to treat high blood pressure coercively? And that is something I perhaps wouldn’t consider right*.

-Resident in psychiatry, female.

##### Protecting patients from social harms

Another example mentioned in the interviews was to protect patients with acute psychiatric disorders from social harms, such as ruined relationships or financial problems. The typical case concerned patients in manic episodes.

*Absolutely, in a manic episode… Yes, yes. To protect them from themselves. […] I don’t find that very complicated because they can ruin so much for themselves and those who are close to them.*

-Resident in psychiatry, female.

#### Promoting autonomy

Another category of reasons for accepting involuntary treatment was that of promoting the patient’s autonomy. Instances concern restoring the patient’s autonomous ability, promoting well-founded decisions, and acting in accordance with what was presumed as the patient’s true will.

##### Restoring autonomous ability

Paradoxical as it may seem, autonomy was mentioned as a possible justification for compulsory treatment. The issue was raised in various circumstances, the main reason being treatment of psychotic episodes that were seen as detrimental to autonomy.

…*well, you could really argue that sometimes when you make decisions about involuntary treatment, at the end of the day it’s about restoring the patient’s autonomy.*

-Specialist in psychiatry, male.

More specifically, improving decision-making capacity was mentioned:

*But the solution is then to treat the psychiatric disorder so that they are able to understand what their best interest is. Or to optimise their decision-making capacity so that they can make a decision.*

-Resident in psychiatry, female.

##### Promoting well-reasoned decisions

Issues relating to autonomy were also mentioned in relation to preventing patients from impulsive actions that might be detrimental to reasoned long-term goals.

*It could be about giving time. For an important decision, like a suicide or so, so that you can make that decision after more reasoning.*

-Resident in psychiatry, male.

##### Respecting the patient’s presumed will

Several respondents brought up the idea that consent to treatment may be presumed despite the patient’s refusal. One idea was that even though a patient might reject treatment, this rejection would not be representative of the patient’s ‘true’ will, *i.e.* what the patient would prefer if not afflicted.

*It could be anything from a patient with psychosis who has a completely different apprehension of the world and does not, at the moment, want medicine. However, I know that when he is better, he will want it, because he wants to be well and get out of this.*

-Specialist in psychiatry, female.

One respondent expressed this in terms of what the patient would want, if they possessed adequate cognitive abilities.

*No, I act in accordance with what I believe [the patient] would want if he had the time and the intellectual and emotional ability to reflect on his situation. Presumed consent.*

-Specialist in psychiatry, male.

#### Safeguarding third party interests

A third category of reasons was reasons relating to third-party interests. This included both preventing danger to others (beside the patient) and relieving relatives of responsibility.

##### Preventing harm to others

One aspect raised by some respondents was danger of harm to others, which was considered a major reason for decisions in favour of involuntary treatment. The examples given all referred to patients with psychotic symptoms. One respondent talked about a psychotic patient who had tried to set fire to her apartment.

*She doesn’t let healthcare personnel in and she doesn’t let social workers in. And maybe she needs another place to live according to me – in order to minimise the risk of relapse into this kind of serious criminality and danger to others.*

-Specialist in psychiatry, female.

##### Relieving relatives of responsibility

One respondent mentioned the interests of patients’ relatives as a relevant factor regarding decisions about compulsory treatment. He argued that relatives should not be left with the responsibility of caring for seriously ill or suicidal patients, since they would lay so much blame on themselves if the patient were to harm him- or herself.

*If you are that worried about a patient. And think that she is, is suicidal or…psychotic so that she doesn’t know what she’s doing. Then… then it would not be ethically justifiable to impose that [responsibility] on the relatives.*

-Resident in psychiatry, male.

#### Reasons against involuntary treatment

A number of situations were described where one or more of the above mentioned reasons for ordering involuntary treatment were present, but where contra reasons could still make the psychiatrist refrain from deciding in favour of involuntary admission.

##### Involuntary treatment as an unwanted exception

One general assertion was that involuntary treatment was problematic and constituted an unwanted exception to standard care.

*Treatment against the will of the patient…I mean that should be seen as a real exception.*

-Resident in psychiatry, male.

##### Avoiding disruption of trust

The main reason against compulsory treatment mentioned was typically the risk of the patient–physician alliance being harmed and patients losing confidence in the psychiatric services if a decision about compulsory treatment were to be made. This was stated as a reason for not ordering involuntary treatment, also in situations where legal criteria would be fulfilled.

…*in the long run there will be a better alliance, she will maintain her trust in psychiatric care, which is the only institution in society that [will be able to] help her if she has a serious mood disorder.*

-Resident in psychiatry, male.

##### Avoiding direct harms of coercion

The reasons against compulsory treatment were often described in terms of harms or negative outcomes of the treatment. The harm could be directly related to limiting patient’s freedom or to the physical coercion that it may involve.

*I wouldn’t say that the problem with mandatory treatment is something abstract. It is that it can be so brutal.*

-Specialist in psychiatry, male.

Infringements of patients’ freedom were seen as a direct harm to be avoided if possible.

*It can really be traumatic for someone to be admitted… 24 h… in a [psychiatric] ward when they don’t want to be.*

-Specialist in psychiatry, female.

##### Respecting self-determination within limits

As noted, autonomy was used as a reason for involuntary admittance and treatment. However, in discussions about involuntary treatment, respect for self-determination was also mentioned as a reason for not ordering involuntary treatment when patients refused care. Respect for self-determination was typically the default position in cases where the immediate risk of harm was seen as low. One respondent told about a case of a patient with bipolar disorder who, after a manic episode, refused treatment with Lithium.

*And that is so frustrating because you know that he would probably do so much better, but he refuses. And so we talked about it a lot and […] He had his reasons for this. And who am I to know better than him?*

-Resident in psychiatry, female

However, it was acknowledged, if the condition were to deteriorate, (involuntary) treatment would still be an option. Another respondent told of a case where she had been consulted as an expert by the court. The patient was diagnosed with bipolar disorder and was deemed to be in a manic state, however the respondent did not agree with the assessment of the attending psychiatrist.

*Then it was better [for the patient] to make these decisions himself, so that he could take the consequences, not blame it on us for locking him up and stopping him… I think he needed to feel that he could take responsibility himself.*

- Specialist in psychiatry, female.

##### Accepting the possibility of rational suicides

As already noted, suicide prevention was mentioned as a major reason for involuntary psychiatric treatment. However, it was also argued that not all possible cases of suicide should be seen as cases for psychiatric care. When asked directly, respondents agreed that so-called ‘rational suicides’ might occur although there were different assertions regarding how common they are. Such suicides were often described as a means of escaping somatic conditions.

*I think that it could be about somatic conditions. Where you may be suffering, you are in terrible pain; you see that your level of functioning is deteriorating. And you don’t have any other way out.*

*-*Specialist in psychiatry, female

However, it was also argued that psychiatrists should be cautious.

*I think that if as a psychiatrist or physician you are to judge whether there are rational reasons, you are into something that is pretty dangerous and hard to tell for any outside person.*

-Specialist in psychiatry, male.

One respondent specifically asserted the right of psychiatric patients to make decisions about when and how to end their lives. The respondent raised this argument in relation to a case where a patient with a psychiatric disorder and a debilitating and painful physical condition had contemplated going abroad in order to complete a physician-assisted suicide.

*And in these discussions, people have hesitated whether to allow patients with serious psychiatric disorders to make decisions about this. And I do not think that should be a hindrance, it should rather be a right.*

-Specialist in psychiatry, female*.*

### Circumstances affecting decisions about involuntary treatment

The second section of categories concerns the circumstances in which decisions about compulsory psychiatric treatment are made.

#### The patient’s social circumstances

It was argued that the patient’s social circumstances, *e.g.* social network and economical situation, also could influence whether an involuntary admission was deemed necessary.

*For instance, I do believe that it [involuntary admission] is more common among people who do not have that much resources and…relatives that take care of them….*

-Resident in psychiatry, male.

#### Legal influence

The relationship between ethics and the law was described in two ways. The law set a basic framework for the ethical deliberations and was often commended by the respondents; however, it was also seen as something open to interpretations by the psychiatrists.

##### Legal demands

Several respondents acknowledged that the law heavily influenced their ethical deliberations. Mostly, the law was seen as commendable, but some of the respondents were also critical of ethical issues often being treated as mere legal issues.

*It becomes so easy to conflate ethics and the law. When you work so much with it. You get used to … to think according to the law.*

-Resident in psychiatry, male.

##### Interpreting the law

However, respondents also acknowledged that the law in itself was not very clear and that in most situations it left room for personal judgments and choices between different alternatives.

*Like I said before: first you think about it ethically: should you try to save her? And if you decide that you will try, then you will have to use the law the way you can.*

-Resident in psychiatry, male.

Another respondent argued similarly that whether or not it was possible to admit a patient involuntarily depended on how the physician chose to present the patient’s symptoms in the involuntary treatment order. In the case discussed by the respondent, the respondent believed that there was a risk of the patient harming herself if she were not admitted. The patient, however, did not accept to stay at the ward.

*And I think that the first criterion is not really satisfied. That there is a serious psychiatric disorder. […] But, anyway I chose to order involuntary treatment. I was thinking that I would be able to get the criterion fulfilled anyway.*

-Resident in psychiatry, male.

#### The possibility of informal coercion

In some cases the patient refused or was reluctant to accept care, but a formal decision about involuntary treatment was deemed inappropriate or not possible. Here, there was a ‘grey zone’ between persuasion and manipulation that could be used to make the patient accept treatment and this functioned as an alternative to formal coercion.

##### Restricting options

One respondent argued that, particularly in somatic care, one way to achieve this was to restrict information about alternatives.

*You don’t create that many alternatives for the patient. [You say] ‘This is…we have to give you this.’ You behave in a way that does not give the patient any alternatives*.

-Specialist in psychiatry, male.

##### Using the law to make the patient accept “voluntary” treatment

Another way to make the patient comply was to merely introduce the possibility of coercive treatment. One respondent gave an example of how this might be carried out:

*Maybe you can put it this way: I have… I am worried about you, and so I am considering whether to order involuntary treatment if you do not agree to be admitted voluntarily.*

-Specialist in psychiatry, female.

#### Healthcare deficiencies

The functioning of health care also influences decision-making regarding involuntary treatment according to the respondents. Here it was argued that decisions about compulsory treatment were made that could have been avoided in a more well-functioning healthcare organisation.

##### Inadequate care environment

It was argued that the atmosphere in the clinical ward affected how patients behaved and how they were treated. This included features such as presence of other distressed patients, attitude and behaviour of the staff and whether or not patients knew the staff from before.

*I think that just coming in to a psychiatric emergency ward creates a number of incentives for coercive measures that would not exist in a calmer and friendlier atmosphere, where the patients know the staff.*

-Specialist in psychiatry, male.

It was also argued that the need for coercive interventions varied from one ward to another, depending on the staff and their approach to the patients.

*For instance, the amount of coercive measures varies, depending on what nurse works there. And that depends on…how you treat the patients.*

-Resident in psychiatry, male.

##### Inadequate resources

When physicians were not able to give patients appointments for return visits, coercive measures were deemed more likely.

*And then it feels like you are using mandatory treatment in order to… make sure the patient gets appropriate follow-up.*

-Resident in psychiatry, male.

## Discussion

In line with the Swedish legislation, the respondents typically saw involuntary treatment as justifiable in cases where the patient had a serious psychiatric disorder and rejected treatment, or where there was a direct risk of self-harm. A commonly mentioned reason against compulsory treatment was the risk of disrupting the doctor-patient relationship and ruining trust in the psychiatric services. In this way, much of the reasoning was based on assumptions and deliberations about consequences, where the physician would aim to make the decision he or she believed benefitted the patient the most short term as well as long term. However, there was a general feeling conveyed in the interviews that involuntary treatment was problematic in itself and something to be avoided. This notion was often implicit in the reasoning and rarely expressed in so many words.

### Suicide and self-determination

When it came to the actual cases described, suicidality was possibly the most important reason mentioned for justifying compulsory treatment. However, the respondents generally agreed that suicides may be rational and not necessarily a consequences of psychiatric disorders, but they disagreed about how common such suicides are. One argued that such cases, albeit theoretically possible, were not clinically relevant, while another respondent claimed to encounter such patients on a regular basis when working as a liaison psychiatrist at a general hospital. The commonly quoted example of what would be a ‘rational suicide’ focused on cases involving somatic disorders or persons at the end of life who were suffering greatly and to whom no relief was available. Thus, the rationality of suicides were primarily discussed in terms of when circumstances and consequences would make it rational for persons to end their lives, not in terms of autonomy or decision-making capacity [46].

One of the respondents stated that patients should have a right to end their lives, including a right to assisted suicide, and that this right should also include patients suffering from psychiatric disorders. Another respondent argued that even though rational suicides exist, psychiatrists should remain cautious about the concept and refrain from declaring any particular instance of suicide, or suicidal ideation, as rational. Thus, although these respondents shared basic factual assumptions about the existence of rational suicides, their statements highlight a normative difference on how healthcare should handle such cases.

### Autonomy considerations

Issues relating to patient autonomy were used as a reason both for and against involuntary treatment. Restoration of autonomy in non-autonomous patients was held forth as a goal for treatment, most notably in cases of psychosis. Respect for personal decisions was held to be a reason against compulsory treatment, mainly in cases of somatic disorders. In general, concerns for patient autonomy focused more on the idea of autonomy as a value to be promoted or restored than as a right to have one’s decisions respected [3]. It was often presumed that patients in need of compulsory treatment were also decisionally incapable [42]. However, lack of decision-making capacity was not broached as an important criterion for when compulsory treatment was justified. Thus, it may be argued that the main ethical principles underlying the respondents’ reasoning are promotion of good (or beneficence) and prevention of harm, rather than respect for autonomy. This is also in line with the findings of Feiring and Ugstad [36].

One interesting line of thought presented by some respondents is the idea of treatment being something that the patient would hypothetically or ideally consent to. In a recent study of psychiatric inpatients, most patients who regained capacity following treatment retrospectively approved of the treatment decisions made on their behalf when decisionally incapable [47]. Thus, it may be argued that even though the patient under current circumstances rejects treatment, the patient’s ‘true’ (*i.e.* mentally healthy or decisionally capable) self would consent to it. This idea relates to an ongoing discussion about the role of authenticity in personal autonomy. It has, in bioethical debate, been argued that healthcare staff may justifiably overrule decisions that are insufficiently authentic in order to protect a patient’s authentic or underlying true interests [3, 48]. In cases where a patient is incapable of making a decision, it may be a useful strategy to try to identify the option that best fits with the patient’s ideals and wishes when decisionally capable. However, it is quite another thing to argue that an otherwise competent patient is insufficiently autonomous because of lack of authenticity. As argued elsewhere, the concept of authenticity seems notoriously hard to define in a way that is both normatively reasonable and applicable in a clinical setting [48]. One problem is that it could more or less always be argued that a patient would have made another decision, if just circumstances were different, or if they had not been ill. However, if any seemingly imprudent decision could be labelled as inauthentic and therefore invalid, the principle of respect for autonomy would lack normative force [2, 48].

### Law and ethics

Sometimes a conflict was described between what the physicians believe is right and the legal demands. It was, however, commonly argued that there is room for different interpretations of the law, and that individual doctors’ judgments played an important role for when and why decisions about involuntary treatment were made. Moreover, several respondents told of situations when the borders between voluntary and involuntary treatment were blurred. Some acknowledged that manipulation could be justifiable if treatment was deemed in the patient’s best interest and compulsory treatment was not a viable option. This reveals an ambiguity: on the one hand it was obvious that the respondents’ own ethical reasoning was closely aligned with the Swedish law, but on the other hand it was generally acknowledged that the law left room for interpretation and personal judgment. With this, the law was sometimes used to make patients accept treatment voluntarily, using an implicit threat of involuntary treatment if the patient would not comply. Possible reasons for this may be that involuntary psychiatric admission is perceived as more stigmatising than a voluntary admission, or that the law may not be applicable in all circumstances. However, even though the physicians may experience such implicit coercion as less a violation of a patient’s freedom than a formal decision about involuntary admission, it may be just as problematic from the point of view of autonomy, or even more so, since the patient is not fully informed about the conditions underlying their admission.

### Institutional factors

Many respondents brought up organisational factors as important for when and how decisions about compulsory care were made. These factors are interesting since they constitute the framework in which the decisions are made, and in many respects they lie outside the physicians’ direct control.

Patients' lack of social support was assumed to increase the likelihood of a decision about involuntary treatment being made. One factor that was not mentioned in the interviews but raised in the study by Feiring and Ugstad was presence of children in the patient’s home. This was mentioned as a reason for involuntary treatment in the Norwegian study [36], and it is also in line with the personal experiences of the authors of this paper that this may play a role for involuntary treatment decisions also in Swedish psychiatric care.

It is noteworthy that several respondents argued that inadequate healthcare organisation and care environment could make decisions about involuntary treatment necessary. Thus, it was claimed that there might be situations where circumstances compel the conscientious physician to make a decision leading to involuntary treatment when this could have been avoided in a better care environment and in organisations with better follow-up routines. As argued elsewhere, issues regarding healthcare organisation and interpersonal treatment are in many cases intertwined [49].

If involuntary treatment is used as a means to cover up deficiencies in standard psychiatric care, the issue of how to improve standard care needs to be raised. Organisational deficiencies can be addressed both at the level of individual hospital organisations and in the broader political context. The issue of how bad interpersonal treatment affects patients may be addressed within the individual healthcare organisation, as well as in professional training. Further studies regarding this would be valuable.

### Limitations

The number of respondents in this study is relatively small and the topics complex, hence it is possible that more interviews would gain further and more nuanced data. However, the obtained data was rich and nuanced, and the last two interviews mainly confirmed findings from the other six interviews, which suggests that our decision to stop at eight interviews was not premature. It is also interesting to point out the similarities between our findings and those in the previously cited study by Feiring and Ugstad [36].

One issue to bear in mind is the character of the issues discussed: questions about the use of coercion, clinical decision making and judgment are sensitive and respondents may adjust their answers to accord with what they perceive as ethically or legally correct. It is also important to note that the qualitative design does not allow for hypothesis testing at a generalised level. Thus, it is not possible to say whether the experiences and viewpoints of the interviewed psychiatrists are representative for Swedish psychiatrists in general.

## Conclusions

In the interviews, ethical deliberations about compulsory treatment were discussed in relation to issues about autonomy and patients’ decision-making capacity. The respondents’ judgments about involuntary treatment were typically in line with Swedish law on the subject. However, it was also argued that the law leaves room for individual judgments when making decisions about involuntary treatment and that informal coercion sometimes would be used as an alternative to a formal decision about involuntary treatment. Much of the reasoning focused on the consequences of ordering involuntary treatment, where risk of harm to the therapeutic alliance was weighed against the assumed good consequences of ensuring that patients received needed care. Cases concerning suicidal patients and psychotic patients who did not realise their need for care were typically held as paradigmatic examples of justified involuntary treatment. However, there was an ambivalence regarding the issue of suicide prevention as it was also argued that the risk of suicide in itself was not sufficient for justified involuntary care, because of the possibility of rational suicides. Yet some respondents claimed that this possibility is not clinically relevant and should be disregarded in actual practice. Respect for autonomy was rarely directly invoked in terms of a right for patients to refuse treatment. However, autonomy was sometimes held as a reason for involuntary treatment in order to promote autonomy or facilitate autonomous decision-making. The respondents also raised issues related to healthcare organisation and the care environment, and it was argued that some decisions about compulsory treatment could have been avoided, given a more patient-oriented healthcare organisation.

## Additional file

Additional file 1:
**Interview guide.**
